# OTUB1 Overexpression in Mesangial Cells Is a Novel Regulator in the Pathogenesis of Glomerulonephritis through the Decrease of DCN Level

**DOI:** 10.1371/journal.pone.0029654

**Published:** 2012-01-18

**Authors:** Yan Zhang, Ruimin Hu, Huijuan Wu, Weina Jiang, Yu Sun, Yan Wang, Yanan Song, Tong Jin, Hongxia Zhang, Xin Mao, Zhonghua Zhao, Zhigang Zhang

**Affiliations:** Key Laboratory of Molecular Medicine of Chinese Education Ministry, Department of Pathology, Shanghai Medical College, Fudan University, Shanghai, People's Republic of China; Sun Yat-sen University Medical School, China

## Abstract

**Background:**

OTUB1 is a member of OTUs (Ovarian-tumor-domain-containing proteases), a deubiquitinating enzymes family (DUBs), which was shown as a proteasome-associated DUB to be involved in the proteins Ub-dependent degradation. It has been reported that OTUB1 was expressed in kidney tissue. But its concrete cellular location and function in the kidney remain unclear. Decorin (DCN) in mesangial cells (MC) is considered to be a potentially important factor for antagonizing glomerulonephritides, and its degradation is mediated by ubiquitination. The aim of this study is to investigate the role of OTUB1 expression in MC and its relationship with DCN during glomerulonephritis.

**Methodology/Principal Findings:**

Using quantitative RT-PCR and Western blot, we demonstrated that OTUB1 mRNA and protein were constitutively expressed in cultured rat MC and found to be upregulated by the stimulation of IL-1β or ATS. OTUB1 overexpression was detected in the mesangial area of glomeruli in some immunocomplex mediated nephritides such as IgA nephropathy, acute diffuse proliferative glomerulonephritis and lupus nephritis by immunohistochemistry. The immunoprecipitation assay demonstrated that OTUB1 interacted with DCN. The overexpression of OTUB1 enhanced the ubiquitination and degradation of DCN in MC.

**Conclusion/Significance:**

These data showed the inflammatory injury could up-regulate OTUB1 expression in MC, which might attribute the promoting effect of OTUB1 on glomerulonephritides to the decrease of DCN level.

## Introduction

Ovarian-tumor-domain-containing proteases (OTUs) are part of the deubiquitinating enzymes (DUBs) family [1], [2]. They have been implicated to play an important role in mediating the processes of proteins ubiquitination and degradation through ubiquitin-proteasome pathway (UPP). DUBs are large groups of cysteine proteases that are classified into six main families, such as UBPs (ubiquitin-processing proteases), UCHs (ubiquitin C-terminal hydrolases), ataxin-3/Josephin domains, OTUs (ovarian-tumour-domain-containing proteases), pathogen-encoded ubiquitin-processing proteases and JAMM (JAB1/MPN/MOV34 metalloenzyme) proteases [3]. DUBs can hydrolyze isopeptide bonds between ubiquitin and folded proteins, remove the ubiquitin or polyubiquitins from target proteins, and interfere the degradation of substrates in the Ub-dependent pathway [4]. DUBs are generally recognized as negative regulator to reverse the process of ubiquitinization [3], [5]. However, DUBs are also found recently to be involved in a multiprotein complex of proteasom to facilitate substrates degradation in ER stress. During the proteolysis by proteasome, the removal of the Ub chain from the substrate by proteasome-associated DUBs such as OTUB1 is a key to allow the passage of the unfolded polypeptide through a narrow constriction into the proteolytic chamber of the proteasome core particle, where proteolysis ensues [6], [7].

One of the most recently recognized DUBs is the OTUs. This family mainly comprises a group of putative cysteine proteases including OTUB1, OTUB2, A20 and yeast OTU1 [2]. All of them have an OTU domain of 130 amino acids that is highly conserved from yeast to mammals [1]. The OTU family is a matter of considerable interest to us due to its conserved sequences in viruses, bacteria, plants, yeast, and humans, and its role in immunity and inflammation [8]. OTUB1 was the first member of OTU family to be confirmed for its deubiquitinating properties. It is located at chromosomal position 11q13.1, and is ubiquitously expressed in human tissues [9]. A structural analysis of OTUB1 shows differences in accessibility to the active site and in surface properties of the substrate-binding regions that may reflect functional diversity in regulatory mechanisms and substrate specificity [2]. Recently, study has shown that YOD1, which is the closest homolog of yeast OTU1, performed as a proteasome-associated DUB to be involved in the ER-associated degradation (ERAD) pathway, which is related to the metabolism of numerous glycoprotein [6], [10], [11]. Therefore, OTUB1 is thought to play an important role in many physiological and pathological processes of human being. The OTUB1 gene product is identified to be involved in the control of cell division and differentiation of the cystoblast into an oocyte and nurse cells [12], [13]. Although widely expressed, OTUB1 was specifically implicated in mediating lymphocyte antigen responsiveness through affecting the stability of the lymphocyte-specific E3 ligase GRAIL (gene related to anergy in lymphocytes) in CD4+ T-lymphocytes [14]. Moreover, OTUB1 was also found in Lewy bodies of the brain on mass spectrometry, and may be involved in the pathogenesis of neurodegenerative disorders [15].

OTUB1 expressing in kidney tissue has been detected by RT-PCR and Western blot [16]. However, its concrete cellular location and function in the kidney are unknown. There are also no relative reports about its relationship with kidney diseases.

Decorin (DCN) is a small proteoglycan composed of a core protein and a glycosaminoglycan chain. It has been shown that DCN has a variety of functions and may interfere the binding of TGF-β to its receptor complex [17]. Forced expression of DCN in human mesangial cells (MC) inhibit the expression of TGF-β1 and Collagen IV [18]–[20]. It plays a critical role in the reduction of ECM deposition in mesangium during glomerulonephritis, inhibition of mesangial cell (MC) proliferation, and impediment of glomerulosclerosis development [21].Therefore, DCN is considered to be a potentially important factor for antagonizing glomerulonephritides.

Recently, the investigation of metabolism and regulation of DCN has been expanded. Our prior report showed that intracellular DCN was degraded by the UPP in MC [22]. Furthermore, on mass spectrometric analysis of immunoprecipitation gels using DCN-antibody, we found that OTUB1was highly expressed in MC (data not shown), which suggested a possible interaction of OTUB1 with DCN. We thus speculated that the deubiquitinating enzyme OTUB1 may mediate the process of DCN degradation in MC. To understand the mechanism of this event would help to elucidate the role of OTUB1 expression and its relationship with DCN during glomerulonephritis.

In this study, we examined OTUB1 expression in rat MC with or without stimulation of inflammatory factors and the ectopic expression of OTUB1 in biopsy tissues of human kidney with some common glomerulonephritides. Further immunoprecipitation studies were performed to investigate the interaction between OTUB1 and DCN in MC. The aim of our study was to test our hypothesis that OTUB1 is implicated in pathogenesis of glomerulonephritides.

## Results

### 1. The expression of OTUB1 in cultured MC

**Figure 1:**
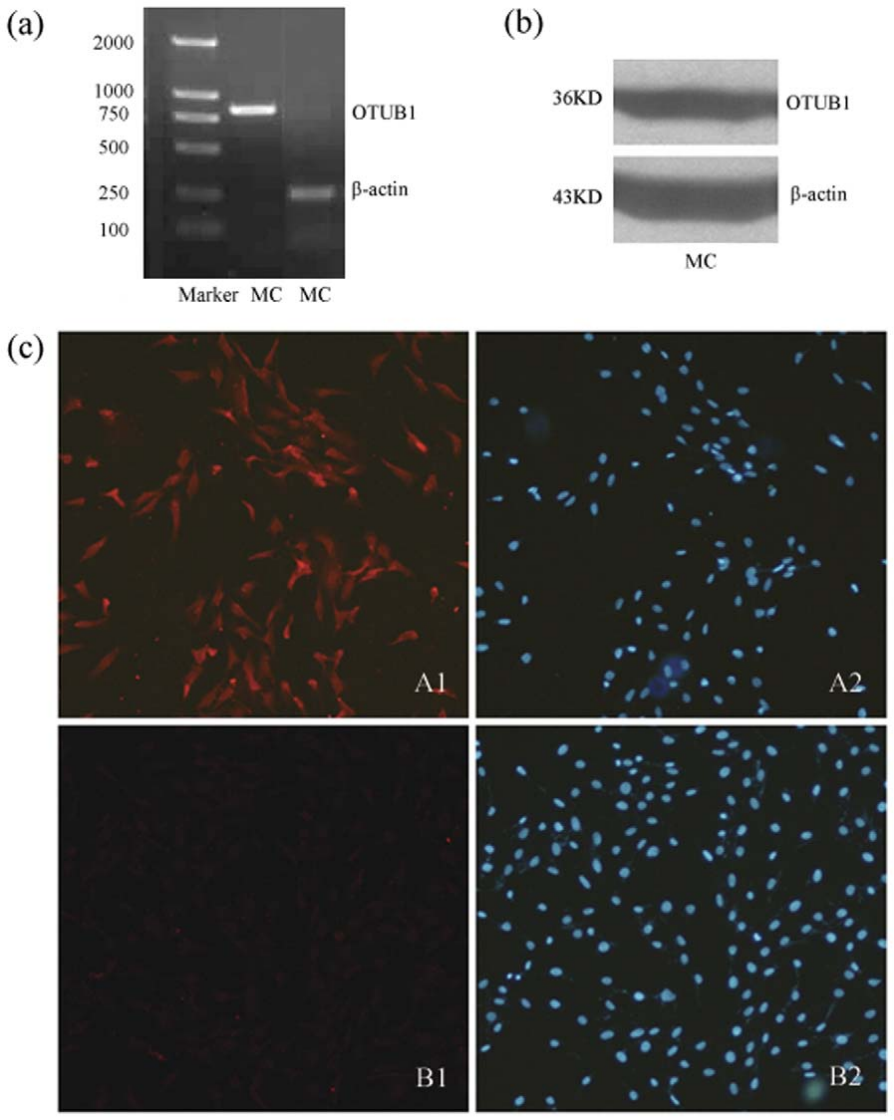
The expression of OTUB1 in MC. (a) RT-PCR analysis of OTUB1 performed on total DNA extracted from MC. (b) Western blot analysis of OTUB1 in MC using anti-OTUB1 antibodies. (c) Immunofluorescence staining of OTUB1 in MC using anti-OTUB1 antibody(A1), Hoechst 33258 staining (A2), isotype control antibody staining (B1) and Hoechst 33258 staining (B2).

In cultured MC, OTUB1 mRNA was found with an amplified fragment of 850 bp by using RT-PCR (Figure 1a), and OTUB1 protein expression was confirmed by Western blot with a molecular weight of 36 kD (Figure 1b). Immunofluorescence detection of OTUB1 was also performed with positive fluorescent signals in the cytoplasm and nuclei of MC compared to the corresponding control (Figure 1c).

### 2. Upregulation of OTUB1 and DCN mRNA in MC were mediated by IL-1β and ATS

**Figure 2:**
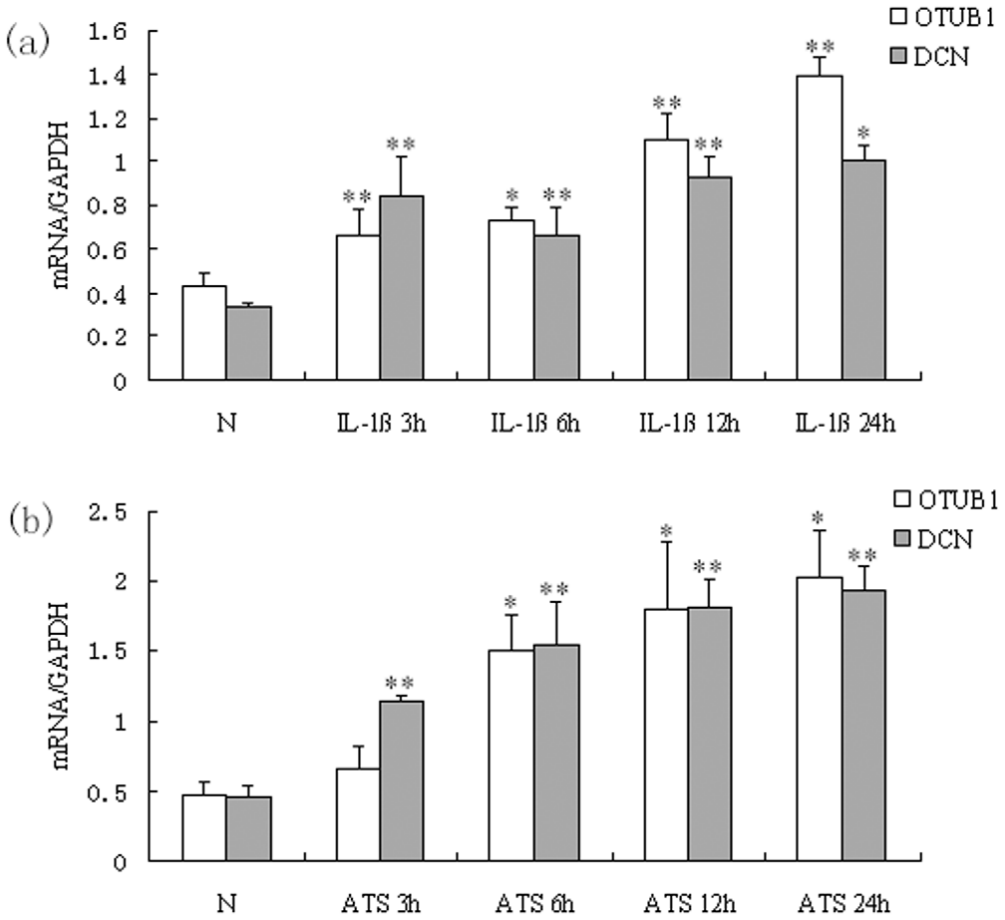
OTUB1 and DCN mRNA were upregulated in MC stimulated with IL-1βand ATS. (a) MC were treated with IL-1β10 ng/mL for 3 h, 6 h, 12 h and 24 h. (b) MC were treated with ATS 30 µL/mL for 3 h, 6 h, 12 h and 24 h respectively. mRNA levels of OTUB1 and DCN in MC were analyzed by quantitative real-time PCR. Besides the group of OTUB1 in ATS treating for 3 h, both OTUB1 mRNA and DCN mRNA significantly increased in all groups treated with IL-1β or ATS compared with normal group (*P<0.05, **P<0.01). Data are presented as mean±standard error (SE) from at least three individual experiments carried out in duplicate.

Quantitative real time PCR showed that OTUB1 mRNA was markedly upregulated in rat MC after administrating of IL-1β for 3, 6, 12 and 24 hours. There was a statistically significant difference between the administrating and normal control groups (p<0.05). To observe the relationship between OTUB1 and DCN in MC, DCN mRNA was also tested synchronously and found to be significantly upregulated in rat MC after exposure to IL-1β for 3, 6, 12 and 24 hours, compared with controls (p<0.05; Figure 2a).

After treatment of MC with ATS for 3, 6, 12 and 24 hours, both OTUB1 and DCN mRNA were significantly increased in administrating groups versus normal groups (p<0.05; Figure 2b), besides the group of OTUB1 in ATS treating for 3 h. This suggested that the IL-1β and ATS may upregulate the expression of OTUB1 in MC.

### 3. The protein expression of OTUB1 and DCN in MC were mediated by IL-1β and ATS

**Figure 3:**
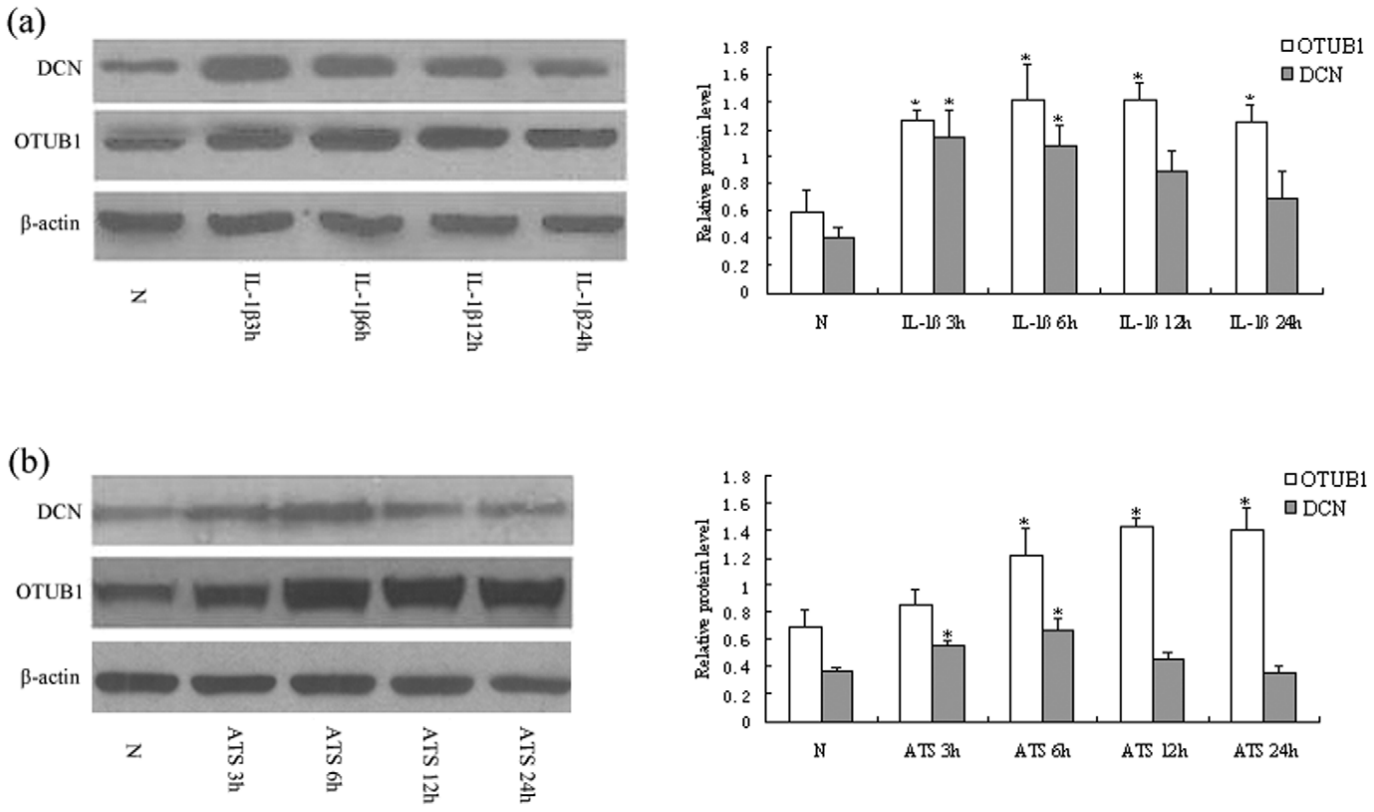
OTUB1 and DCN expression mediated by IL-1β and ATS. (a) MC were treated with IL-1β 10 ng/mL for 3 h, 6 h, 12 h, 24 h, respectively. Western blot analysis showed that OTUB1 expression increased with IL-1β at 3 h, 6 h, 12 h and 24 h, while the increased DCN expression induced by IL-1β was found at 3 h and 6 h, which were significantly higher than that of normal controls. The expression of DCN then decreased quickly at 12 h and 24 h. (b) MC were treated with ATS 30 µL/mL for 3 h, 6 h, 12 h and 24 h. OTUB1 expression was significantly higher in ATS treated cells at 6 h, 12 h and 24 h, while DCN expression markedly increased at ATS 3 h and 6 h compared to normal controls. However, the DCN expression declined again in 12 h and 24 h. (□) OTUB1/β-actin; (▪ )DCN/β-actin. (*P<0.05; **P<0.01). Data are presented as mean±standard error (SE) from at least three individual experiments carried out in duplicate.

We continued to detect the protein level of OTUB1 and DCN in cultured MC after treatment with IL-1β and ATS. Western blot analysis showed that OTUB1 expression increased in cells exposed to IL-1β at 3, 6, 12, and 24 hours (p<0.05, Figure 3a), which was consistent with the expression of their mRNA. However, increased expression of DCN induced by IL-1β was found at 3 and 6 hours, significantly different from that in cells of health controls (p<0.05), then its expression decreased quickly at IL-1β 12 and 24 hours. There was no significant difference of DCN expression at cells of IL-1β 24 h compared with health control cells (p>0.05) (Figure 3a).

The same pattern occurred in MC treated with ATS. OTUB1 expression was significantly higher in ATS treated cells at 6, 12, and 24 hours, while DCN was higher at 3 and 6 hours compared to cells of health controls (p<0.05 ), and again declined in 12 and 24 hours (Figure 3b). These results demonstrated that IL-1β or ATS could upregulate the protein level of OTUB1 in MC. In contrast, both factors could downregulate the protein level of DCN after a transient response of up-expression in the early stages. It suggested that the mechanism of regulation for the two proteins in MC might be different post-transcriptionally.

### 4. Upregulation of OTUB1 in MC with glomerulonephritides

**Figure 4:**
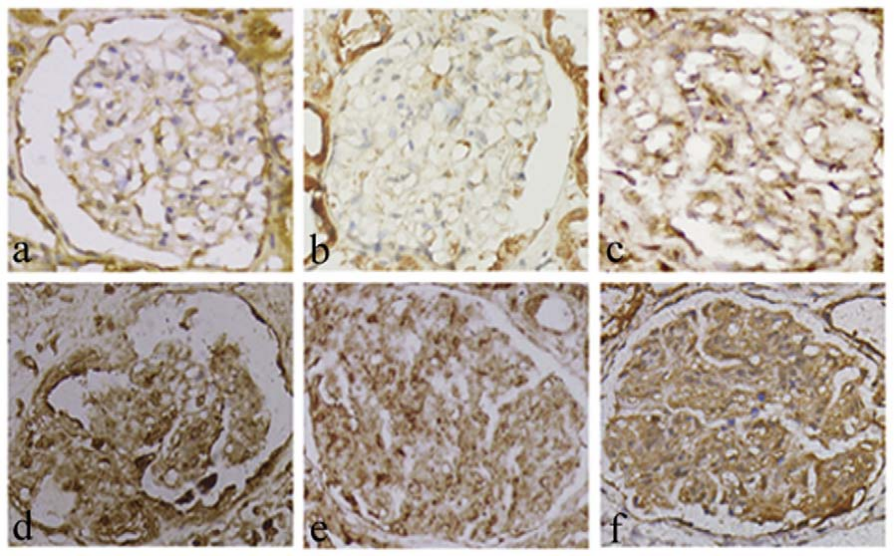
OTUB1 expression in glomerular during some golmerulonephritides was detected by immunohistochemistry. (a) Normal glomerulus, there is few positive staining in golmerulus; (b) minimal change disease; (c) membranous glomerulonephritis, there is minor positive in the mesangium at (b) and (c); (d) IgA nephropathy, moderate positive in the mesangium; (e) acute proliferative glomerulonephritis; (f) lupus nephritis. The increased positive staining was seen in mesangium at (d), (e) and (f). In addition, the tubular epithelial cells are partially diaminobenzidine positive. Hematoxylin is used as the nuclear counterstain. (ABC immunochemistry, ×100).

55 samples were acquired from kidney needle biopsy, including 8 cases from healthy kidney tissue (distant from kidney tumor), 8 minimal change disease (MCD), 7 membranous glomerulonephritides (MGN), 10 mesangial proliferative type of IgA nephropathy (IgAN), 12 acute diffuse proliferative glomerulonephritides (APGN), and 10 lupus nephritis, subtype IV (LN-IV). Immune-reaction staining demonstrated that OTUB1 expression was diversely present in nephropathy samples, but with few positive cells in the normal glomeruli. The degree of OTUB1 expression in the mesangial region of the glomeruli differed among the various types of glomerulonephritides. The average optical density in the glomerulus was 0.004506 in MCD, and 0.004028 in MGN; both were not significant different from that in normal kidney tissue (0.002096) (p>0.05). In contrast, the average optical density was 0.03412 in IgAN, 0.065412 of the APGN glomerulus, and 0.088363 of the LN-IV glomerulus, all of which were significantly higher than that in normal kidney tissue (p<0.01) (Table 1). OTUB1 immunostaining was localized mainly in the mesangial area, in which the brown granules were distributed diffusely, which consisted with the morphological features of dendritic MC. Moreover, some of the parietal epithelial cells of Bowman's capsule, crescent and part of the tubular epithelium were OTUB1 positive. It was demonstrated that OTUB1 expression was upregulated in MC of diseased glomeruli, which correlated with the type and intensity of pathological changes in various glomerulonephritides (Figure 4).

**Table 1:** Quantitative analysis of OTUB1 immunoreactivity in glomerulonephritides.

groups	n	mean density(IOD/AOI)
Normal	8	0.002096±0.00034
MCD	8	0.004506±0.002153
MGN	7	0.004028±0.002089
IgAN	10	0.03412±0.003823**
APGN	12	0.065412±0.020209**
LN	10	0.088363±0.034724**

Immune staining of OTUB1 was quantitatively assessed on the mean density (ratio of integrated optical density (IOD) to area of interest (AOI) per glomerulus in normal kidney tissue and some nephropathy tissues. The expression of OTUB1 was statistically significantly higher in IgA nephropathy, acute diffuse proliferative glomerulonephritides and lupus nephritis than that in normal kidney tissue (**P<0.01).

### 5. OTUB1 overexpression downregulated expression of DCN in MC

**Figure 5:**
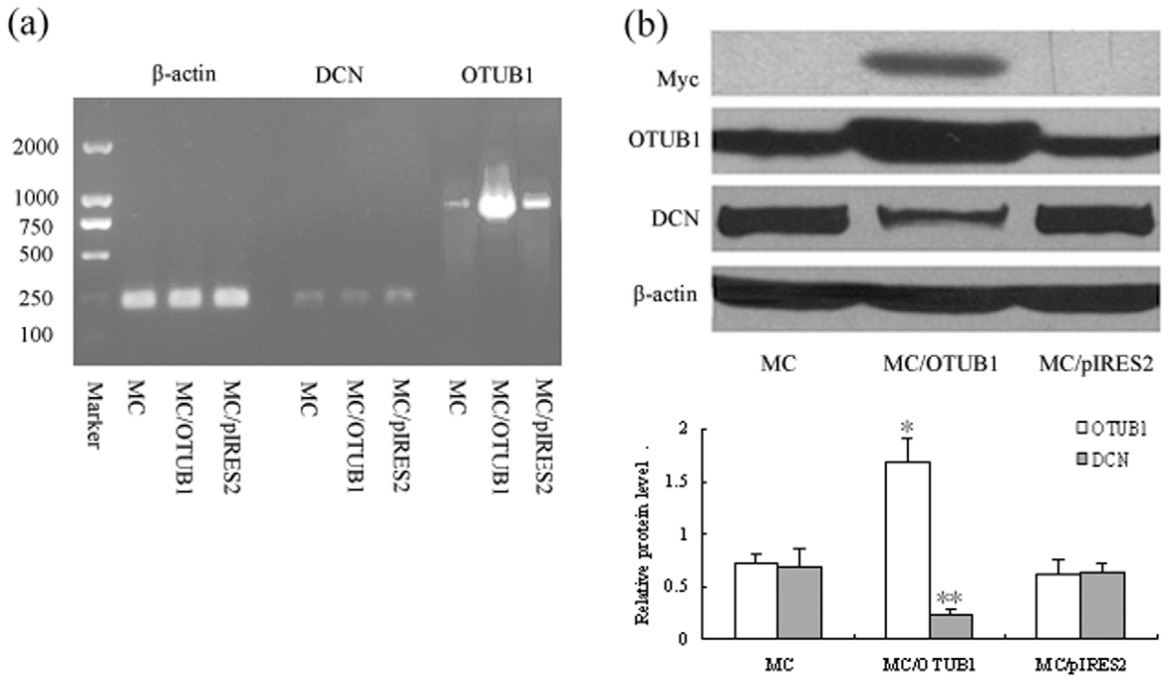
Overexpression of OTUB1 downregulated the expression of DCN in MC. (a) mRNA expression of OTUB1 in MC after OTUB1 gene transfection was assessed by RT-PCR. Lane 2, 5, 8 are normal mesangial cells (MC) as control, Lane 3, 6, 9 are pIRES2-EGFP-OTUB1-myc stable transfected MC (MC/OTUB1), Lane 4, 7, 10 are vacant vector pIRES2-EGFP transfected MC (MC/pIRES2). The mRNA expression of OTUB1 increased in lane 9. (b) Protein expression of OTUB1 and DCN were assessed by Western blot. Lane 1 is normal mesangial cells (MC) as negative control, Lane 2 is pIRES2-EGFP-OTUB1-myc stable transfected MC (MC/OTUB1), Lane 3 is vacant vector pIRES2-EGFP transfected MC (MC/pIRES2). Overexpression of OTUB1 was seen in lane 2 with myc labeling positive. Meanwhile, the DCN expression significantly decreased after OTUB1 gene transfection in lane 2. (□) OTUB1/β-actin; (▪ )DCN/β-actin. (*P<0.05; **P<0.01). Data are presented from at least three individual experiments carried out in duplicate.

To study the role of OTUB1 in regulating the level of DCN in MC, we constructed eukaryotic expression plasmid pIRES2-EGFP-OTUB1-myc and stable transfected vector into cultured rat MC by cationic lipid mediator. The RT-PCR and Western blot results showed that the positive cell clones markedly overexpressed OTUB1 mRNA and protein, including myc tag protein (Figure 5a–b). Meanwhile, the DCN protein levels were also obviously lower in OTUB1 transfected MC than that in normal control cells (Figures 5b). The data indicated that OTUB1 overexpression could induce a decrease of the DCN level in rat MC.

### 6. OTUB1 and DCN immunoprecipitation with increasing the ubiquitination of DCN in MC

**Figure 6:**
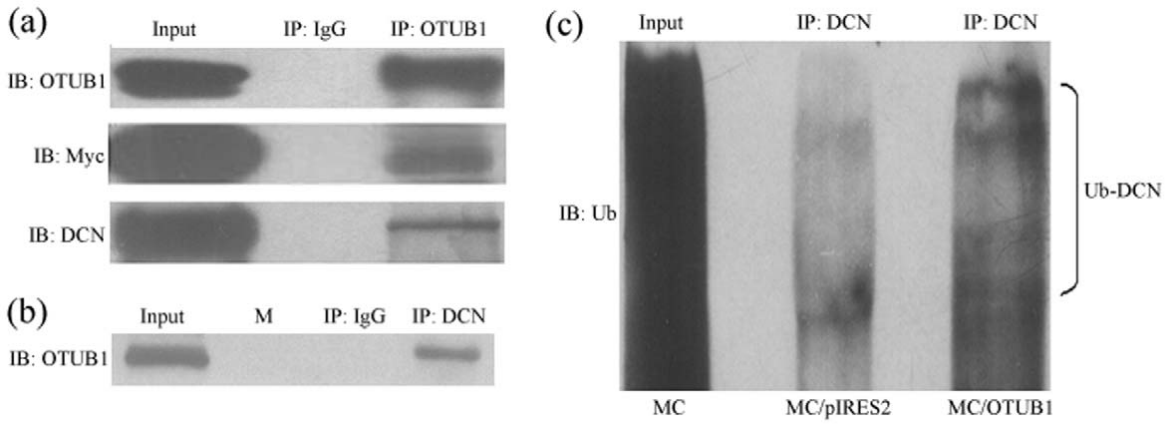
OTUB1 interaction with DCN in MC concomitant with increase of DCN ubiquitination. (a) MC was stable transfected with pIRES2-EGFP-OTUB1-myc. Then we performed IP with OTUB1 antibody and analyzed by SDS-PAGE using antibodies against the OTUB1, myc and DCN. (b) Reciprocal IP with anti-DCN antibody was analyzed using OTUB1 antibody. Lysate from MC (input) was used as a positive control, isotype matched nonspecific IgG as negative control. Lane 2(M) represents marker. (c) Cell lysates were subjected to immunoprecipitation with anti-DCN antibody followed by immunoblotting with an anti-ubiquitin (Ub) antibody. Lane 1 is normal mesangial cells (MC), cell lysates were analyzed by immunoblotting. Lane 2 is vacant vector pIRES2-EGFP transfected MC (MC/pIRES2) as control, Lane 3 is pIRES2-EGFP-OTUB1-myc transfected MC (MC/OTUB1), overexpression of Ub was seen in lane 3.

To further study the link between OTUB1 and DCN in MC, immunoprecipitation assays were performed with an anti-OTUB1 antibody and blotted with anti-OTUB1, anti-myc, and anti-DCN antibodies in OTUB1 transfected MC. As shown in Figure 6a (top bands), the OTUB1 and myc bands were seen in MC. While the nonspecific IgG was negative, the cellular total protein was the positive control. Reprobing the same membrane with anti-DCN antibody confirmed the specificity of OTUB1 combining with DCN (Figure 6A, lowest band). Conversely, in reciprocal immunoprecipitation with anti-DCN, the OTUB1 was also detected in depositing protein linking with DCN (Figure 6b). Then, the ubiquitination of DCN was determined by anti-ubiquitin antibody after DCN immunoprecipitation. As shown in Figure 6c, the level of ubiquitinated DCN were significantly higher in OTUB1 transfected MC than that in vacant vector transfected MC. These results indicate that OTUB1 does interact with DCN and promote the process of DCN ubiquitination and degradation.

## Discussion

Here we report, for the first time, OTUB1 expression in cultured rat MC with or without inflammatory factor stimulation by using quantitative real time PCR and Western blot, and in human MC of glomeruli from various nephritides by using immunohistochemistry. Balakirev demonstrated that OTUB1 expression was relatively higher in the kidney [16]. It remains uncertain whether OTUB1 is expressed in glomeruli. In our study, constitutive expression of OTUB1 was shown, and upregulation of OTUB1 mRNA and protein level induced by IL-1β and ATS were observed in cultured MC. ATS can interact with antigens on the cell membranes of MC to form immune complexes, which subsequently activate complement in fresh serum to assemble the sublytic C5b-9, leading to immune injury of cells in vitro [23], [24]. Therefore, it was confirmed that cytokines and immune stimulation could promote OTUB1 upregulation in MC. Consisting with the cell test, OTUB1 overexpression was also observed in mesangial area in some immunocomplex-mediated nephritides such as IgAN, APGN and LN. These data suggested that OTUB1 upregulation may relate to the pathological changes of glomerulonephritides.

Recently, the OTU family has attracted attention for its role in innate immunity and inflammation of cells [8], [14], [25], [26]. The OTU Characterized by the presence of ovarian tumor domain-containing sequences acts as general deubiquitinases. It can hydrolyze ubiquitin and ISG15 from cellular target proteins, antagonize the antiviral effects of ISG15, which enhance the susceptibility to Sindbis virus infection in vivo [27]. A20 (also known as tumor necrosis factor alpha-induced protein 3), another member of OTU family [28], is a unique protein that exerts both NFκ-B inhibitory and anti-apoptotic activities, and is now recognized as a key regulator in inflammation and immunity [8], [26], [29]. Mice deficient for A20 are hypersensitive to TNF and die prematurely due to severe multi-organs inflammation and cachexia [30]. Recent genetic studies demonstrate a clear association between several mutations in the human A20 locus and immunopathologies such as Crohn's disease, rheumatoid arthritis, systemic lupus erythematosus, psoriasis, and type 1 diabetes [31]. However, the mechanism of OTUs mediation in these diseases has still not been fully elucidated.

The pathogenesis of many primary and secondary glomerulonephritides is also related to disorders of immune and inflammatory response. Here we have found that OTUB1 ectopic expression was related to MC injury and pathological changes of glomerulonephritides. Therefore, we speculated that OTUB1 may play an important role in the development of glomeruli diseases. In this work, we not only confirmed the overexpression of OTUB1 in diseased glomeruli, but also demonstrated that OTUB1 could interact with DCN and promote DCN degradation. Since DCN is an important antagonizing factor for glomeruli inflammation, it is suggestion that OTUB1 may be another novel regulator involved in the pathogenesis of glomerulonephritis.

We previously determined that glycoprotein DCN in MC was regulated via the UPP for its degradation [22]. Here we further demonstrated that OTUB1 overexpression induced by gene transfection or by inflammatory stimulation could increase the DCN ubiquitination and proteolysis in MC. The Schlieker' lab reported that another OTUB1-like protein YOD1 was a proteasom-associated deubiquitinating processing factor in the context of protein dislocation from the ER and degradated by proteasomal proteolysis [6]. YOD1 is a constituent of a p97 complex for proteasome and it displays a strong ability to deconjugate K48 linked Ub chains [9], [28]. Ubiquitination is a highly dynamic process carefully controlled by opposing Ub-conjugating and deconjugating activities. In the case of the 26S proteasome, deubiquitination is needed to remove the impediment of attached Ub chains to allow the substrates get into the proteolytic core particle. The otubain core domain of YOD1 is necessary and sufficient for catalytic activity hydrolyzed K48-linked poly- and di-Ub chains degradation of truncated substrate. A dominant negative YOD1 variant stalled the proteolysis and caused accumulation of ubiquitinated substrates [6]. So it coincides with the fact that OTUB1 may serve as proteasome-associated DUB to exert important function for a deubiquitinating activity in the process of DCN degradation.

DCN can neutralize TGF-β1 bioactivity and inhibit the development of glomerulonephritis and glomerulosclerosis [32], [33]. DCN administration has been advocated as a potential antagonist against nephropathies because of the relative deficiency of DCN and relative excess of TGF-β1 existing in glomerulonephritis [18], [19]. Deficiency of DCN has been shown to enhance progressive nephropathy in diabetic mice [32]. Some reports showed that DCN expression increased in rat diabetic kidneys and in glycogen-stimulated cultured MC, suggesting a possible compensatory response to antagonize local TGF-β1 activity [33]. But, DCN is absent in chronic glomerulosclerosis and interstitial fibrosis in renal diseases [34], [35]. Therefore, the question remains: what is the change of DCN during the development of chronic glomeruli diseases? In some glomerulonephritides and severe diabetic glomerulosclerosis, increased DCN concentration was found in the urine. Failure to detect increased glomerular proteoglycan quantity during the development of nephropathy could be partly explained by assuming that they are secreted into the mesangial matrix, partly to form complexes with TGF-β1 and partly to be cleared via the vasculature or the urinary tract [36], [37]. Furthermore, it was interesting to note in our work that DCN was transiently upregulated in cultured MC under IL-1 or ATS stimulation, and then returned quickly to baseline (Figure 3), while DCN mRNA was still kept at a high level. It may imply that the loss of DCN was caused by post-transcriptional proteolysis. These phenomena revealed that a part of the intracellular DCN protein might be quickly degraded during MC injury. It was further validated by the experiment that overexpression of OTUB1 increased the ubiquitination of DCN in MC. We might thus attribute the promoting effect of OTUB1 on glomerulinephritides to the decrease of DCN level.

In summary, we demonstrated that OTUB1 was expressed in MC in vitro and in vivo. OTUB1 expression was upregulated in MC after inflammatory stimulation. There was a relationship between the OTUB1 upregulation and pathological changes of glomerulonephritides. OTUB1 could interact with DCN in MC. The overexpression of OTUB1 promoted DCN ubiquitination and degradation, which may contribute to the pathogenesis of glomerulonephritides. Thus, OTUB1 revealed to be a possible novel regulator during glomerulonephritides, which would facilitate degradation of DCN and enhance the vicious circle characterized by increased TGF-β1 production and matrix deposition in developing glomerulosclerosis. Our work also presented an example of OTUB1 as a proteasome-associated DUB to mediate proteasomal proteolysis.

## Materials and Methods

### Antibodies and reagents

Mouse anti-DCN was purchased from R&D Systems (Minneapolis, MN, USA). Rabbit anti-OTUB1, mouse anti-myc monoclonal antibody, mouse anti-β-actin monoclonal antibody, and IL-1β were purchased from Sigma-Aldrich (St. Louis, MO, USA). mouse anti-ubiquitin monoclonal antibody and Protein G PLUS-Agarose was purchased from Santa Cruz Biotechnology, Inc. (Santa Cruz, CA, USA). Plasmid Midiprep Kit, Opti-MEM, Lipofectamine 2000, and Trizol reagent were purchased from Invitrogen (Carlsbad, CA, USA). The Super Signal West Pico stable peroxide solution enhanced chemiluminescence (ECL) system and the bicinchoninic acid (BCA) Protein Assay Reagent Kit were obtained from Pierce Biotechnology (Rockford, IL, USA). All other reagents were obtained from Sangon Biological Engineering Technology & Service Co. Ltd. (Shanghai, China).

### Cell culture and treatment

MC were prepared from the cortex of male Sprague–Dawley rat kidneys [38]. The cells were utilized between passages 6 and 10 and maintained in RPMI 1640 medium supplemented with 10% newborn bovine serum (NBS) at 37°C in a humidified atmosphere containing 5% CO2/95% air. MC were treated with different reagents containing 10 ng/mL of recombinant rat IL-1β or 30 µl/ml of rabbit ATS (anti-Thy1 serum) for 3, 6, 12, and 24 hours, respectively. ATS was made in our lab as described in a prior paper, which could cross-react with antigens on the cell membrane of the MC *in vitr*o [39].

### Total RNA extraction and quantitative real-time RT-PCR analysis

Total RNA was extracted from the lysates of rat MC using Trizol reagent. Reverse transcription (RT) was carried out using a PrimeScript RT reagent Kit at 37°C for 15 minutes and 85°C for 5 seconds. Real time quantitative RT-PCR was performed using a SYBR Premix Ex Taq™ Kit, in a total reaction volume of 20 µl containing 10 µl of SYBR Green RT-PCR master mix, 2 µl of cDNA template and 0.4 µM of each target-specific primer designed to amplify a part of each gene. After PCR, a melting curve analysis was performed to demonstrate the specificity of each PCR product as a single peak. The relative amounts of OTUB1, DCN and GAPDH mRNA were calculated by comparison with standard curves [40]. Changes in the mRNA expression level were calculated following normalization with GAPDH. The sequences of OTUB1 used were: sense: 5′-GCGACCACATCCACATCA-3′, anti-sense: 5′-TAGGACCATTTACAACCACA GA-3′. The sequences of DCN used were: sense: 5′-TGGCTAAGTTGGGATTGA-3′, anti-sense: 5′-CTGAAGGTGGATGGCTGT-3′. The sequences of GAPDH used were: sense: 5′-AACGGATTTGGTCGTATTG-3′, anti-sense: 5′-GGAAGA TGGTGATGGGATT-3′. The sizes of the amplified fragments were 263 bp, 294 bp. and 208 bp for OTUB1, DCN, and GAPDH, respectively. All samples were run three times. RT-PCR: RNA Extraction and reverse transcription are the same as above. The conditions for PCR were hot-start (95°C) for 2 min, followed by 30 cycles of amplification (denaturation at 95°C for 30 s, annealing at 58°C, 55°C and 56°C for OTUB1, β-actin and DCN for 30 s, respectively and extension at 72°C for 30 s). The final extension was at 72°C for 7 min. The sequences of OTUB1 used were: sense: 5′- GCCCTCGAGATGGCGGCGGAGGAACCTC-3′, anti-sense: 5′- GCGGATCCTCACAGATCCTCTTCTGAGATGAGTTTTTGTTCTTTGTAG-3′; and the sequences of β-actin used were: sense: 5′- AGGATGCAGAAGGAGATTACTGCC-3′, anti-sense: 5′- AAAACGCAGCTCAGTAACAGTGC-3′. The sequences of DCN used were: sense: 5′-TGGCAGTCTGGCTAATGT-3′, anti-sense: 5′- ACTCACGGCAGTGTAGGA -3′. The sizes of the amplified fragments were 850, 248 and 199 base pair for OTUB1, β-actin, and DCN respectively. Final PCR products were electrophoresed in a 1% agarose gel.

### Protein isolation and Western blot analysis

Cell pellets was lysed in cold cell lysis buffer (pH 7.4) (50 mM Tris-HCl, 150 mM NaCl, 1 mM EDTA, 1% Triton X-100, 10% glycerol, 0.2 mg/mL NaN3, 1 mg/mL pepstatin A, 1 mg/mL aprotinin, 1 mg/mL leupeptin, and 1 mmol/L phenylmethylsulfonyl fluoride) for 30 minutes on ice. Then, 60 µg of protein was loaded with 5×SDS loading buffer and resolved by (10%) SDS-polyacrylamide gel electrophoresis (PAGE), then transferred onto a PVDF membrane (Millipore, Eschborn, Germany). The membrane was blocked with 5% non-fat milk solution in room temperature for 1 hour, then immunoblotted with OTUB1 polyclonal antibody (1∶50 dilution), DCN monoclonal antibody (1∶1,000 dilution), Ubiquitin monoclonal antibody (1∶1000 dilution ) and β-actin monoclonal antibody (1∶1,000 dilution). Detection by enzyme-linked chemiluminescence was performed according to the manufacturer's protocol (ECL; Pierce Biotechnology). The housekeeping protein β-actin was used as a control. Results were analyzed quantitatively using Gelpro32 (Media Cybernetics, Shanghai, China). Each experiment was repeated at least three times.

### Immunoprecipitation

Precleared cell lysates (1 mg total protein) were immunoprecipitated with 1 µg of antibodies against either OTUB1 or DCN plus 30 µL of Protein G Sepharose beads, after adjusting the volumes to 0.5 ml with NET buffer (50 mM Tris–HCl, pH 7.4, 150 mM NaCl, 0.1% Nonidet P (NP)-40, 1 mM ethylene diamine tetra-acetic acid, 0.25% gelatin, 0.02% sodium azide, 1 mM PMSF, and 1% aprotinin), and incubated overnight at 4°C. The beads were centrifuged at 2000 G for 5 minutes at 4°C and washed three times with 500 µL NET buffer, and once with PBS. Immunoprecipitated proteins were eluted with 5×SDS loading buffer and resolved by standard SDS–PAGE. The following steps were the same as for Western blotting.

### Immunofluorescence

For immunofluorescence experiments, cells were seeded onto the glass coverslip and fixed in paraformaldehyde. After a brief washing with PBS buffer, the fixed cells were incubated with anti-OTUB1 (1∶100) antibody and nonspecific isotype control antibody (1∶100) for 1 hour at 37°C, followed by incubation with TRITC-conjugated secondary antibody at 37°C for 45 minutes. Photos were taken by fluorescence microscope.

### Immunohistochemistry

Renal needle biopsies were collected at the nephrosis laboratory, Department of Pathology, Shanghai Medical College, Fudan University, in accordance with our institutional ethics guidelines. Paraffin sections (4 µm) of biopsy tissue were deparaffinized, endogenous peroxidase was quenched using 3% H_2_O_2_, and antigen retrieval was performed by microwave in a 10 mmol/L citrate buffer, pH 6.1. Non-specific binding was blocked using 5% normal sheep serum for 30 minutes at 37°C. Sections were then incubated with a rabbit polyclonal anti-OTUB1 antibody (1∶100) at 37°C for 1 hour, then overnight at 4°C. Immobilized antibodies were detected by biotinylated secondary antibody (ABC assay Kit; Vector Laboratories, Orton Southgate, UK). Diaminobenzidine (DAB) was used as the chromogen substrate and hematoxylin stain was the nuclear counterstain. The primary antibody was replaced by PBS or normal rabbit serum (1∶100) as negative control.

Immunohistochemistry of the OTUB1 staining was assessed using Image-Pro Plus 6.0 software (Media Cybernetics). At least five glomeruli were selected from each section under microscopy. the positive area inside a glomerular was calculated with the total OTUB1-positive area in global capillary tufts divided by the whole glomerulus area. The mean absorbance of the OTUB1 position per glomerulus was calculated for each nephritis group. The absorbance of OTUB1 position was defined as the integrated optical density (IOD) in the global capillary tufts divided by the whole glomerulus area (area of interest, AOI).

### OTUB1 Stable transfection on rat MC

The pIRES2-EGFP-OTUB1-myc plasmid was amplified in *E. coli* DH5.α and the authenticity was verified by sequencing. MC (1×10^6^ cells) was seeded in six well plates with 70–80% confluence. The cells were washed twice with PBS, then three times with Opti-MEM before transfection. The plasmid DNA (10 µg) of pIRES2-EGFP-OTUB1-myc and 10 µL of Lipofectamine 2000 were incubated in 250 µL Opti-MEM for 5 minutes at room temperature and then mixed for another 30 minutes at room temperature. The 500 µL complex was added into the culture dishes and the cells were incubated at 37°C for 6.5 hours, followed by the addition of 2 ml 10% fetal calf serum/DMEM medium. After 3 days, stable clones were selected in the presence of 0.4 mg/ml Geneticin (G-418, Sigma). After 3 weeks, positive clones were selected by both Western blotting and RT-PCR analysis after culture expansion. Control cells were transfected with an empty vector pIRES2-EGFP alone.

### Statistical analysis

Statistical analysis was performed using SPSS software (Chicago, IL,USA). The Student's t-test was used for analysis of paired data in the *in vitro* assays. The one-way ANOVA test was used to assess the differences among multiple groups in the *in vivo* assays. A p-value of less than or equal to 0.05 was regarded as statistically significant. Data were presented as the mean ± standard deviation (SD) of triplicate experiments. The density of the bands on the Western blots was quantified by densitometry and analyzed by a Gel-Pro Analyzer from Media Cybernetics (Silver Spring, MD, USA).
